# Editorial: Immune-Related Non-Communicable Diseases in Africa

**DOI:** 10.3389/fimmu.2021.816257

**Published:** 2022-01-11

**Authors:** Mohamed-Ridha Barbouche, Brian Stephen Eley

**Affiliations:** ^1^ Department of Immunolgy, Pasteur Institute of Tunis, Tunis, Tunisia; ^2^ Tunis El Manar University, Tunis, Tunisia; ^3^ Paediatric Infectious Diseases Unit, Red Cross War Memorial Children’s Hospital, Capetown, South Africa; ^4^ Department of Paediatrics and Child Health, University of Cape Town, Capetown, South Africa

**Keywords:** immune-related, non-communicable, diseases, Africa, research

The African continent has a long-standing tradition of research in communicable diseases. Indeed, several national, regional, and international agencies are investing in understanding the pathogenesis of and immunity to infection, particularly those diseases which are endemic to the continent and responsible for substantial morbidity and mortality, notably HIV/AIDS, tuberculosis, and malaria. More recently, interesting efforts have been made to encourage and support research in the field of neglected tropical diseases. In contrast, with few exceptions African immunologists are less involved in researching other immune-mediated diseases such as allergy, cancer, autoimmunity, inborn errors of immunity (IEI), and auto-inflammation despite an expansion of the burden of these diseases on the African continent, commensurate with the epidemiological transition from infectious diseases to chronic, non-communicable diseases. Public health authorities are reporting a surge of non-communicable diseases. Their particularities in African populations are interesting challenges worthy of investigation.

To dissect the immunological mechanisms underlying several of these immune-mediated non-communicable diseases and highlight their specificities in Africa were the aims of this Research Topic. The frontiers between communicable and non-communicable diseases also require exploration, such as host genetic susceptibility to infection, and the interplay between allergy and parasitic infection.

Ten articles were included in this Research Topic and classified into the following categories: six original and not original research manuscripts (Elhaj Mahmoud et al., Tahiat et al., Engelbrecht et al., Harrison et al., Sibanda et al., Ait Ssi et al.); three case reports (Rais et al., Snen et al., Belaid et al.) and one review (Ghouzlani et al.).

Three original research papers explored immune mechanisms underlying autoimmune and rheumatological conditions. Sibanda et al. investigated systemic sclerosis in Zimbabwe, an autoimmune disease that is rarely reported in Africa. In a large cohort of 240 patients, they showed that specific autoantibodies are biomarkers of the condition and can precede overt disease. Autoantibodies also determine disease phenotypes and vary between racial groups. Elhaj Mahmoud et al. studied the pro-inflammatory activity of Wnt5a in rheumatoid arthritis tissue-derived fibroblast-like synoviocytes. They showed that this activity is enhanced *in vitro* in the presence of SFRP5 through mechanisms involving the inhibition of TCF4 and LRP5 expression. These findings may contribute to the identification of potential therapeutic targets. In another contribution, Harrison et al. described features of the arthropathy rarely observed in children with advanced HIV infection, which may be linked to immune suppression where multiple immune aberrations contribute to loss of self-tolerance.

Molecular aspects of IEI were addressed in two original studies and two case reports. Tahiat et al. completed the first systematic evaluation of autoimmunity and autoantibody profiles in Algerian patients with IEIs. Of approximately 300 IEI patients screened for 54 different autoantibodies, 32% had detectable autoantibodies, a significantly higher prevalence than the healthy control group. Predictably, the prevalence of autoantibodies was highest among those patients with diseases of immune dysregulation. An interesting benefit of systematic screening was the identification of several asymptomatic patients with transglutaminase IgA antibodies who on further investigation were shown to have early celiac disease. In Africa, next generation sequencing technologies are not widely available for evaluating patients with suspected IEI. The study of Engelbrecht et al. demonstrated the utility of whole exome sequencing and/or targeted gene panels in 80 patients with suspected IEI. Although molecular diagnoses of IEI were confirmed in 31%, the impact of molecular testing was far greater, influencing the management of two-thirds of the patients. The introduction of routine molecular diagnostic testing is an important priority for African countries. This will improve patient care as shown in this study, increase our understanding of epidemiology of IEI in Africa particularly in many countries with limited or no access to routine immunological diagnostics, and enhance the continent’s capacity to contribute to elucidating the molecular pathogenesis of IEI. Novel case reports by Rais et al. and Belaid et al. reveal phenotypic commonality between IPEX and ALPS and expands the spectrum of hyper-IgE syndrome to include selective interleukin-2 receptor common gamma chain defects, respectively.

Immunity to cancer in the African context is addressed in one original research paper and one review, both focusing on the immunological microenvironment of glioma, a common primary brain neoplasm. Ait Ssi et al. analysed the tumour microenvironment using transcriptomic data from patients with glioma. Unsupervised, hierarchical clustering after enrichment analysis enabled the identification of two patient groups, one characterized by immune cells with anti-tumour immune potential and the other enriched by immunosuppressive immune cells. The timely review by Ghouzlani et al. complemented the findings of the Ait-Ssi study and described immune checkpoints and checkpoint inhibitors used by glioma cells to regulate the immune response and evade immune destruction. While these molecules are potential immunotherapeutic targets, a major consideration is the development of immunotherapies that will cross the blood brain barrier in concentrations capable of blocking immune checkpoint inhibitors used by glioma cells to escape the immune response.

Finally, the paper by Snen et al. described the treatment of a complicated case of allergic bronchopulmonary aspergillosis (ABPA) in a patient with underlying asthma. This report draws attention to the difficulty of diagnosing ABPA and the importance of early diagnosis and effective treatment to circumvent the long-term morbidity of the condition.

In conclusion, this Research Topic documented immunological perspectives on a range of non-communicable diseases, demonstrating the potential of African immunologists to contribute to this interesting area of research and clinical practice.

## Author Contributions

M-RB and BE did edit the Research Topic and did write the editorial. All authors contributed to the article and approved the submitted version.

## Conflict of Interest

The authors declare that the research was conducted in the absence of any commercial or financial relationships that could be construed as a potential conflict of interest.

## Publisher’s Note

All claims expressed in this article are solely those of the authors and do not necessarily represent those of their affiliated organizations, or those of the publisher, the editors and the reviewers. Any product that may be evaluated in this article, or claim that may be made by its manufacturer, is not guaranteed or endorsed by the publisher.

